# Quantitative analysis of choroidal and retinal thickness and blood flow in a rat model of lens-induced myopia using OCTA

**DOI:** 10.1371/journal.pone.0357037

**Published:** 2026-08-27

**Authors:** Qi Zhang, Zhiyi Wu, Jinghan Zhang, Haoyan Zhang, Jiaxin Yuan, Tao Zuo, Lei Zhao

**Affiliations:** 1 Liaoning University of Traditional Chinese Medicine, Shenyang, China; 2 Department of Ophthalmology, the Second Affiliated Hospital of Liaoning University of Traditional Chinese Medicine, Shenyang, China; Sanmenxia Central Hospital, Henan University of Science and Technilogy, CHINA

## Abstract

**Purpose:**

The aim of this study was to use optical coherence tomography angiography (OCTA) to quantitatively analyze the changes in the thickness of the choroidal and retinal layers as well as the alterations in intraretinal blood flow in lens-induced myopia (LIM) rats, and to provide a basis for mechanistic studies of myopia models from the perspective of the choroid and the retina.

**Methods:**

We conducted LIM modeling in rats and set up a normal control (NC). After 8 weeks of modeling, use a high-resolution anterior and posterior segment biometric OCTA to measure the axial length, choroidal thickness, and the thickness of the inner, outer, and entire retinal layers in two groups of rats, and quantify changes in blood flow in the inner layer of the retina. The results were analyzed using t-test.

**Results:**

Compared with NC, LIM rats had longer axis, thinner choroid thickness, thinner inner, outer and whole retinal thickness, and lower blood flow density in inner retina. The differences were statistically significant at most measurement points.

**Conclusions:**

OCTA revealed three myopia hallmarks: axial elongation, choroidal/retinal thinning, and retinal hypoperfusion. These findings enhance clinical understanding of myopic morphological changes and provide mechanistic insights into choroidal-retinal contributions. OCTA’s noninvasive high-resolution enables longitudinal monitoring of ocular structural/vascular changes, offering a basis for diagnosing and preventing high-myopia retinopathy.

**Translational relevance:**

This study used OCTA to non-invasively quantify retinal and choroidal structure and blood flow abnormalities in a myopia animal model, providing translatable imaging biomarkers for the early clinical screening of retinal lesions associated with high myopia.

## Introduction

In recent years, the prevalence of myopia has risen sharply worldwide and has reached epidemic proportions. It is predicted that by 2050, half of the global population will suffer from myopia [1].Myopia, particularly high myopia, may lead to macular degeneration, choroidal neovascularization, retinal detachment, and various other conditions that are significant contributors of visual impairment and blindness [2]. Since the pathogenesis of myopia has not been fully clarified and our comprehension of myopia-related fundus lesions is incomplete, there are currently no effective treatments for myopia. Therefore, investigating the mechanisms associated with the initiation and development of myopia remains a a focal point of research [3]. Research has found that the characteristic manifestations of myopia are an elongation of axial length (AL) and the mechanical stretching and thinning of the retina and choroid [4,5]. Furthermore, numerous research reports indicate that retinal perfusion also decreases in highly myopic eyes [2]. As a result, researchers have focused on the axial length, the choroid, and the retina of myopic eyes, and they hope to obtain data on the thickness of the retina and choroid, as well as blood flow of the retina in myopic animal models to simulate the results in humans, in order to further in-depth basic experimental research.

Optical Coherence Tomography Angiography (OCTA) uses near-infrared light to provide high-resolution cross-sectional images of ocular tissues that allow analysis of the spatial relationships between layers in the posterior segment of the eye. It is a noninvasive, reproducible, noncontact, noninvasive ophthalmic imaging technique, and in contrast to OCT, OCTA allows for high-resolution imaging of the vascular network, which is of high practical value.Therefore, researchers often utilize OCTA to distinguish and evaluate the blood flow and structures of the choroid and retina layers for more profound studies on myopic eye structures [6]. This study employed the OCTA for Anterior and Posterior Segment Biometries (Intalight Saiwei Ruyi Panoramic Eye OCT) to quantitatively analyze the axial length, choroidal and retinal thickness, and alterations in inner retinal blood flow in a myopic rat model with lens-induced myopia, using rats as a model. This study aims to provide a basis and foundation for exploring the relationship between myopia and changes in the retina and choroid, and for future experiments in humans.

## Materials and methods

### Experimental animals and grouping

Twenty SPF-grade healthy SD rats without eye diseases, aged 21 days, weighing between 65g and 75g, consisting of an equal number of males and females, were purchased from Liaoning Changsheng Bio-technology Co.,with an experimental animal license (SCXK (Liaoning) 2023---0004). The experimental animals were raised in the Animal Experiment Center of the Second Affiliated Hospital of Liaoning University of Traditional Chinese Medicine.Prior to the commencement of the study, approval was granted by the Animal Ethics Committee of the Second Affiliated Hospital of Liaoning University of Traditional Chinese Medicine (No. LZYY240403).

All animals were housed under normal barrier laboratory conditions, with free access to food and water, at a room temperature of 22 ± 1°and a humidity of (55 ± 5)%. The light or dark cycle was set to alternate every 12 hours. Animal health and behavior were observed daily. Following a 7-day acclimation period with feeding, animals were examined and any with eye abnormalities were excluded. The rats were then randomly divided into two groups: normal control (NC) and lens-induced myopia (LIM). In the normal control group, the right eye was designated as the experimental eye, while the left eye served as the within-animal control, with neither eye receiving any treatment, and both were kept under conventional housing conditions for 8 weeks. In the lens-induced myopia group, the right eye was the experimental eye, where a model of lens-induced myopia was established, and the left eye served as the within-animal control without any treatment, and both were also housed for 8 weeks.

### Establishment of a lens-induced myopic model

After anesthetizing the myopic lens-induced group rats by intraperitoneal injection of sodium pentobarbital (20 g/L, 0.2 mL/100 g), a -5D myopic defocus lens with a diameter of approximately 1.7 cm was sutured around the skin tissue of the right eye using 5−0 polyester braided thread, completely covering the right eye without pressing on the eyeball, not affecting normal blinking, and leaving the left eye naturally exposed. The periocular skin was disinfected with iodine before the operation, and after the operation, tobramycin dexamethasone eye drops were administered to the suture site between the lens and eyelid skin to prevent infection. The eyes of the rats were observed daily, and if the lens was found to be loose or dirty, it was reinforced and cleaned in a timely manner to ensure that the optical defocus state remained unchanged. If myopic refractive media opacity was observed during the experiment, the rat would be excluded. After continuous covering of the right eye of the rats for 8 weeks, subsequent measurements were taken.

### Axial length measurement

**Fig 1:**
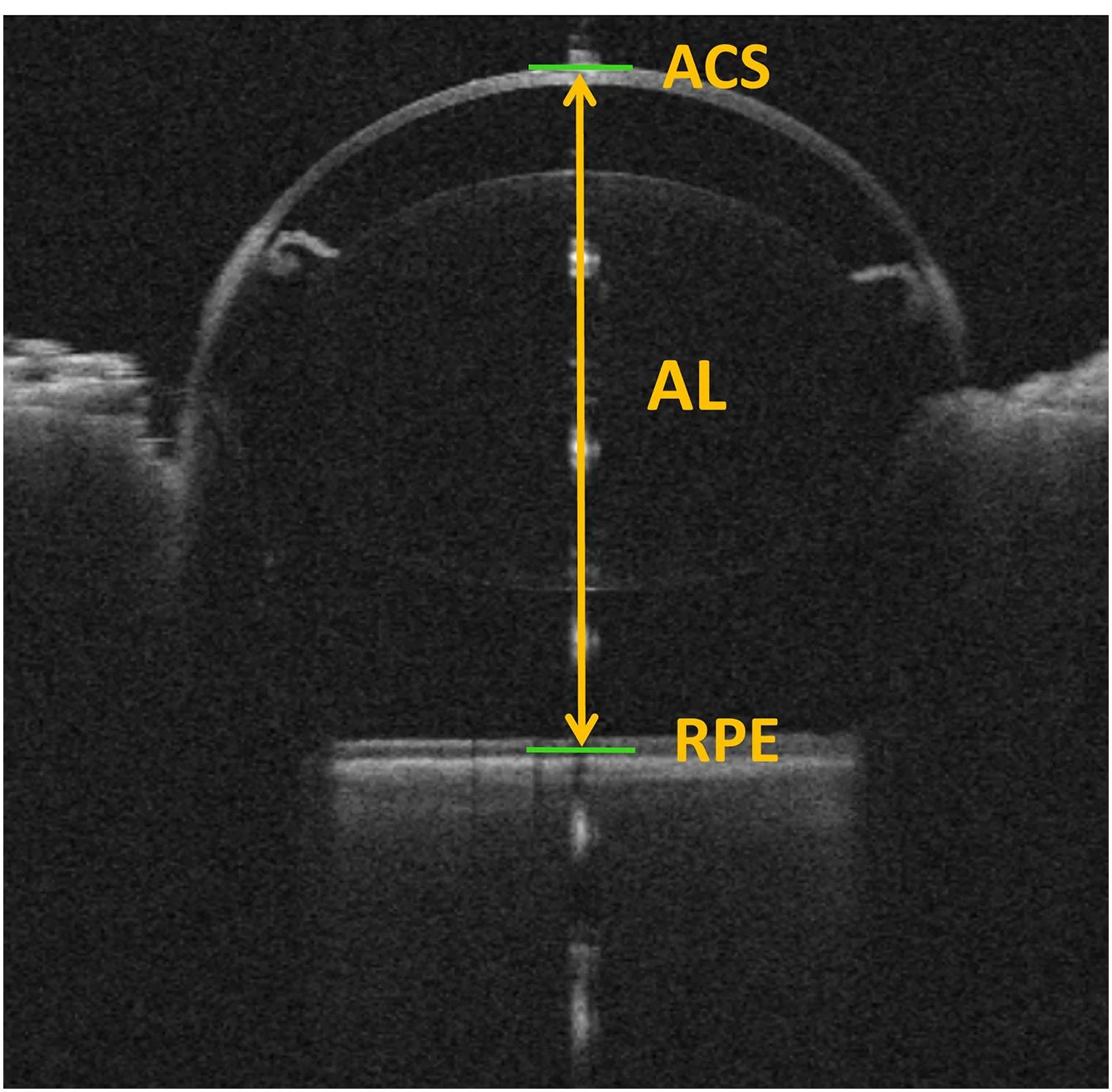
Legend for axial length measurement.

After 8 weeks of modeling, rats were anesthetized with an intraperitoneal injection of sodium pentobarbital (20 g/L, 0.2 mL/100 g), and their pupils were dilated employing compound tropicamide eye drops (drug specification: 1 ml∶5 mg). Utilizing the Intalight Saiwei Ruyi Panoramic Eye OCT, the AL was measured with the instrument’s built-in scanning mode AS Cube 16 mm × 16 mm. AL represents the distance from the high reflective signal of the anterior corneal surface (ACS) to the high reflective signal of the retinal pigment epithelium (RPE) layer. Each rat was measured three times by the same experimenter, with the results being averaged. The method for Axial Length Measurement is shown in Fig 1.

### The thickness of choroidal and retinal

Utilize the 33 Line R16 scan pattern to capture choroidal images, obtaining horizontal cross-sectional images through the optic disc. Divide into three concentric circles with diameters of 2 mm, 4 mm, and 6 mm, placing them at the center of the optic disc, add navigation lines, and measure the choroidal thickness (CT) at six points on the nasal and temporal sides at 1000, 2000, and 3000μm, which is the distance from the RPE to the inner edge of the sclera.

**Fig 2:**
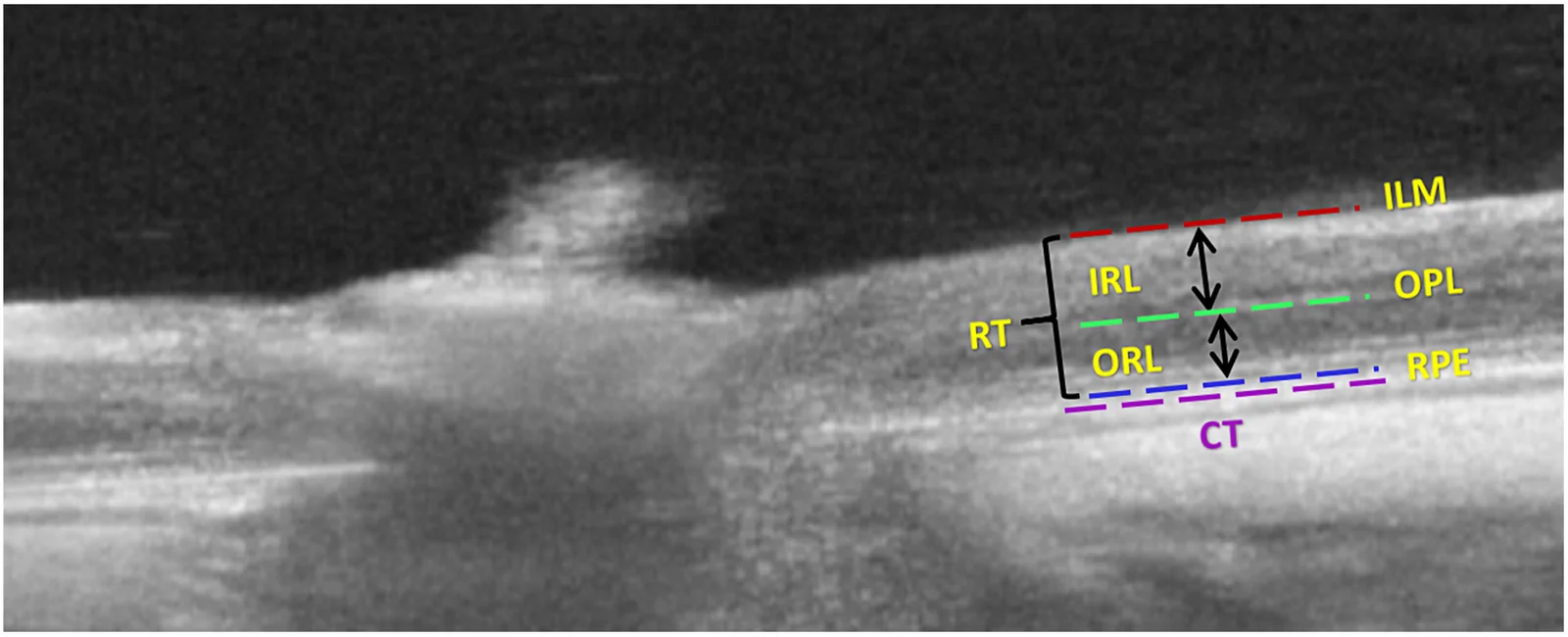
Localization of layers in the choroid and retina.

Capture retinal images using the 33 Line R16 scan pattern, obtaining horizontal cross-sectional images through the optic disc. Divide into three concentric circles with diameters of 2 mm, 4 mm, and 6 mm, placing them at the center of the optic disc, add navigation lines, and measure the thickness of the inner, outer, and entire layers of the rat retina at six points on the nasal and temporal sides at 1000, 2000, and 3000μm. The inner retinal layer (IRL) is from the inner limiting membrane (ILM) to the outer plexiform layer (OPL), the outer retinal layer (ORL) is from the inner edge of the OPL to the RPE, and the entire retinal thickness (RT) refers to the distance from the ILM to the inner edge of the RPE. The method for retinal layer segmentation and thickness measurement is shown in Fig 2.

### Retinal blood flow density measurement

**Fig 3:**
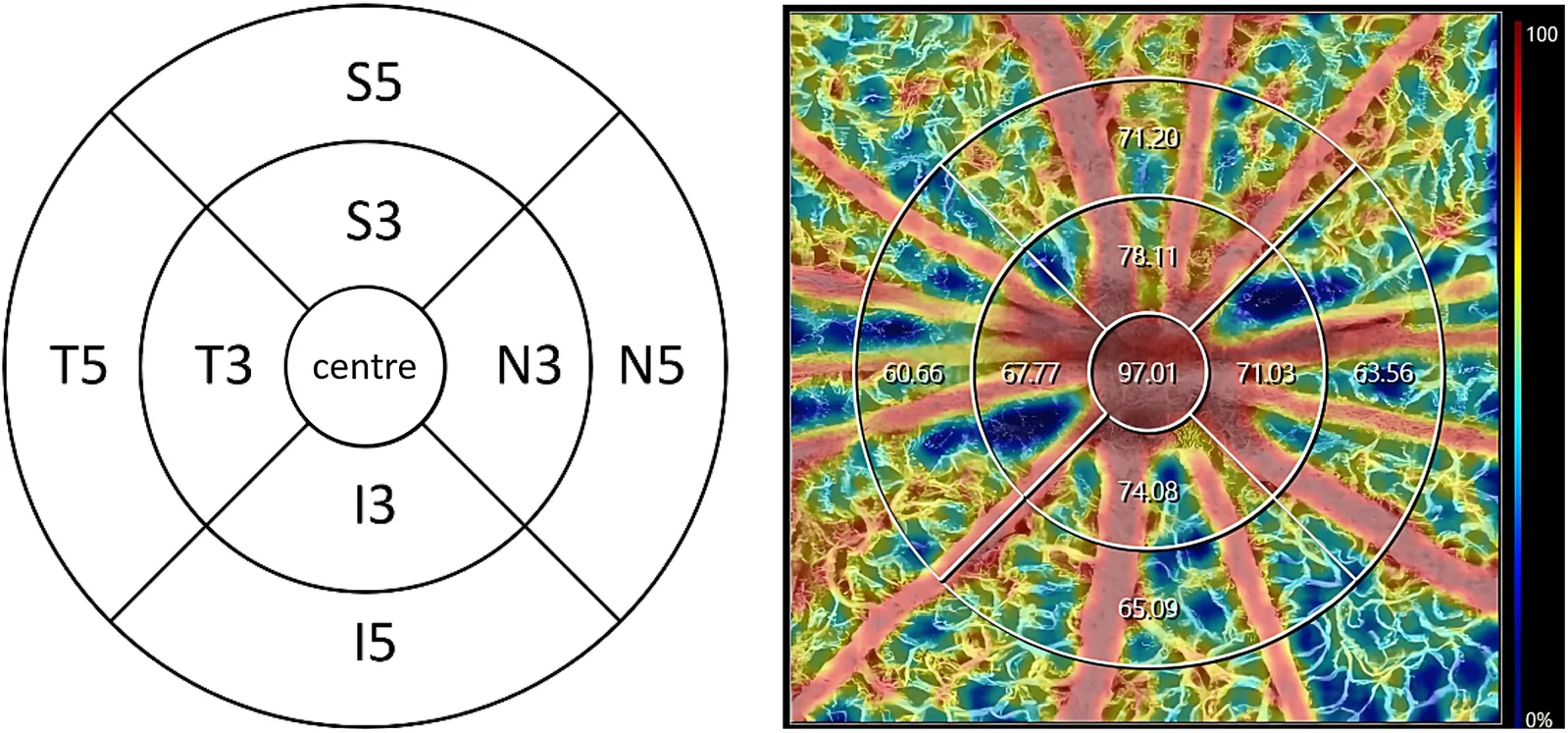
Schematic of the 9 regions of the optic disc (right eye) and Schematic diagram of retinal inner layer blood flow partitioning.

Capture retinal blood flow density images using the Angio 6 × 6 512 × 512 R4 scan mode, and divide them into three concentric circles with diameters of 1 mm, 3 mm, and 5 mm, corresponding to the central, inner ring, and outer ring areas, respectively. Next, use four radial lines to divide the inner and outer ring areas into temporal, superior, nasal, and inferior regions, resulting in a total of nine segments, as shown in Fig 3. Retinal blood flow density is calculated as the ratio of the projected area of blood vessels to the projected area of the specified region on the retinal plane, expressed as a percentage. All measurements were performed by one researcher between 14:00 and 16:00 to minimize differences to mitigate changes in biological rhythms.

(Central area: diameter of 1 mm; T3, S3, N3, I3 are the inner ring areas of the optic disc with a diameter of 3 mm, located on the temporal, superior, nasal, and inferior sides respectively; T5, S5, N5, I5 are the outer ring areas of the optic disc with a diameter of 5 mm, located on the temporal, superior, nasal, and inferior sides respectively.)

### Euthanasia method

The rats were fasted for at least 12 hours prior to euthanasia. After all tests are completed, animals were deeply anesthetized via an intraperitoneal injection of sodium pentobarbital at a dose of 0.2g per 100g body weight. Anesthetic depth was assessed by the absence of pedal and palpebral reflexes. Once a surgical plane of anesthesia was confirmed, animals were euthanized by cervical dislocation performed by trained personnel. Death was confirmed by cessation of respiration and lack of reflex responses. All efforts were made to minimize animal suffering and to use the minimum number of animals necessary to achieve the scientific objectives of the study.

### Statistical analysis

Data was statistically analyzed using SPSS 25.0 software, with all data presented as mean ± standard deviation ($\overset{―}{x}\pm s$). Independent samples t-test was used for comparison between two groups, with a *P* < 0.05 considered statistically significant.

## Results

After modeling for 8 weeks, there were no deaths in either group of rats, and no signs of opacity in the refractive media were observed, no exclusions were necessary.

### The change of AL

**Fig 4:**
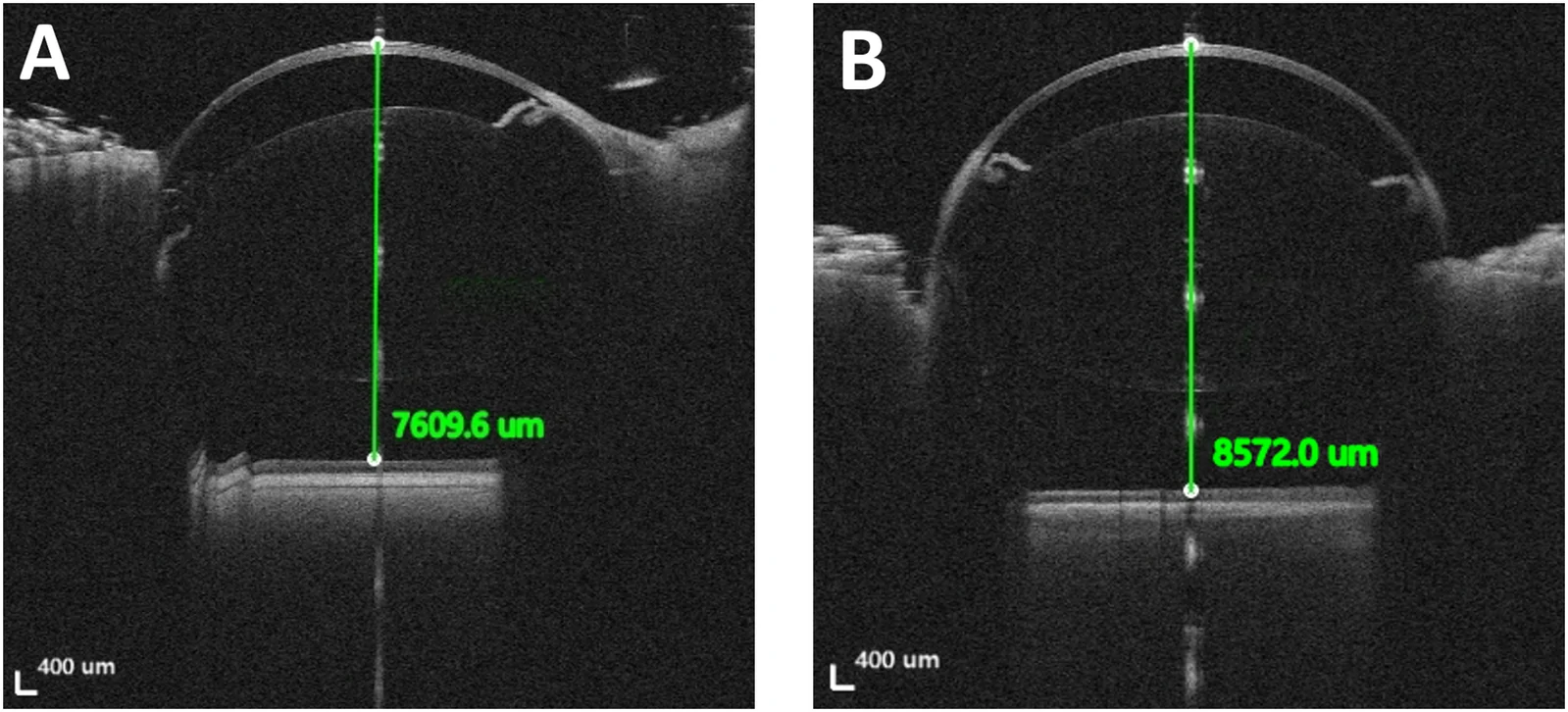
Image of the right eye axis of rats after modeling. (Fig 4-A is for NC rats, Fig 4-B is for LIM rats).

Prior to modeling, the AL of the right eye in both groups of rats were: NC (5607.04 ± 10.24) μm, LIM (5602.91 ± 9.97) μm, with no statistically significant difference (*t* = 0.29, *P* = 0.78). The AL of the left eye in both groups of rats were: NC (5602.22 ± 5.55) μm, LIM (5603.76 ± 19.37) μm, with no statistically significant difference (*t* = 0.08, *P* = 0.94). After 56 days of modeling, the AL of the right eye in NC rats increased to (7624.46 ± 20.26) μm, whereas in LIM rats, it reached (8590.42 ± 23.71) μm. Compared with NC, the LIM group exhibited a significant increase in the AL of the right eye, with a statistically significant difference (*t* = 30.97, *P* < 0.01). This is illustrated in Fig 4. The AL of the left eye in LIM rats increased to (7639.14 ± 30.45) μm. Compared with the left eye in LIM, the right eye exhibited a significant increase in AL, with a statistically significant difference (*t* = 24.65, *P* < 0.01).

### The thickness of choroidal and retinal

**Fig 5:**
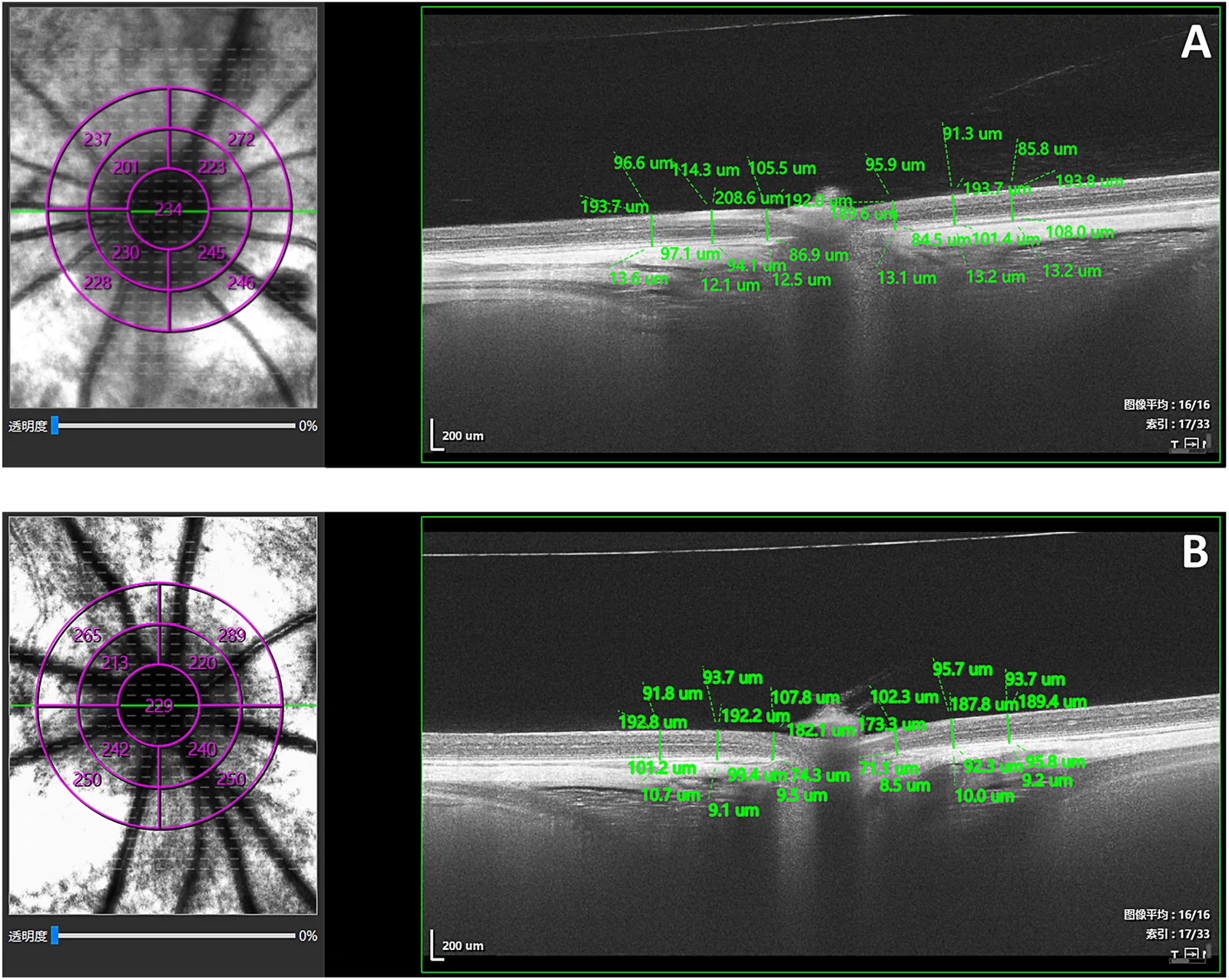
Thickness of the choroid and each layer of retina in the right eye of rats after modeling. (Fig 5-A for NC rats, Fig 5-B for LIM rats).

**Fig 6:**
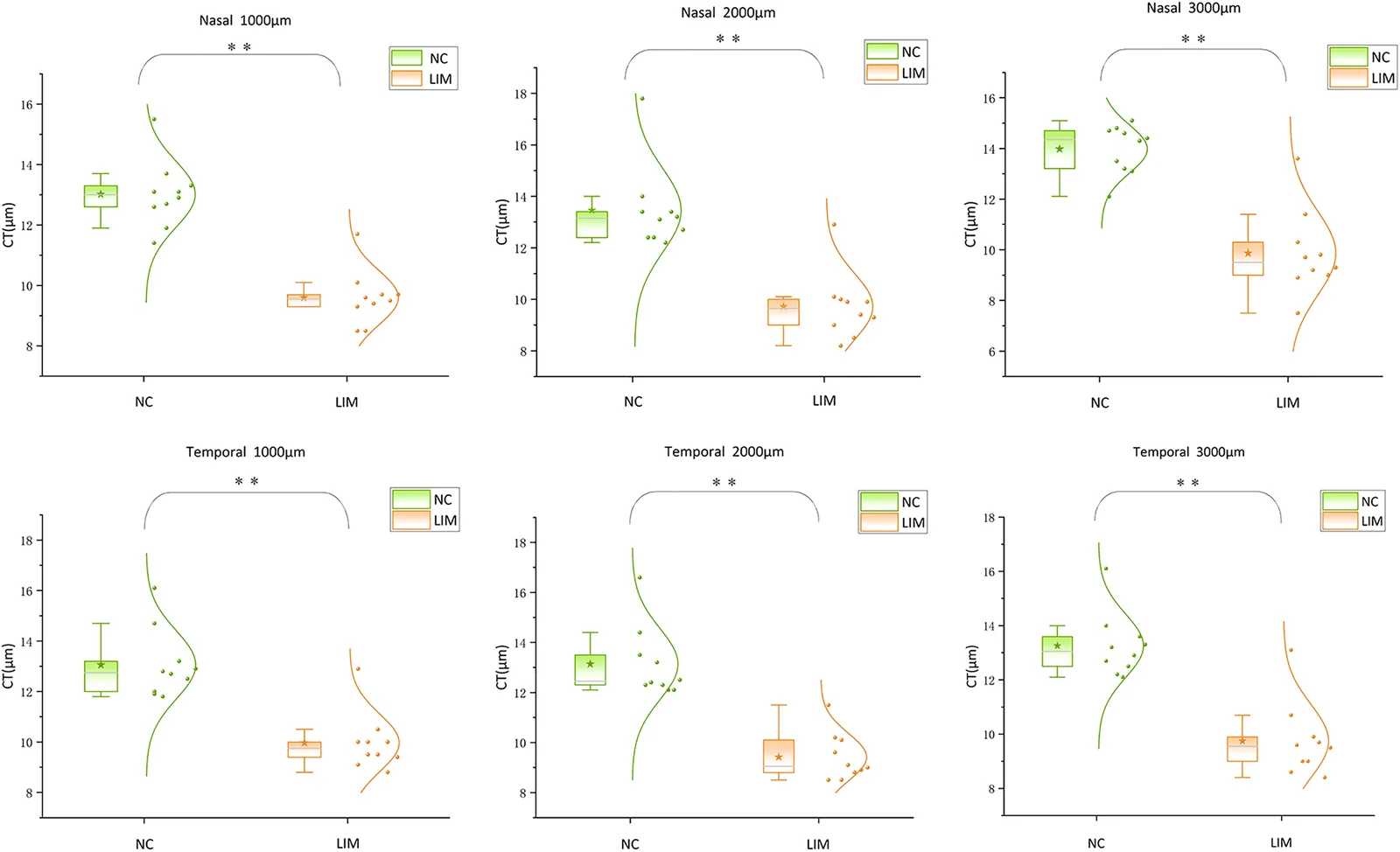
Comparison of CT of right eye between NC and LIM(μm). (At 1000μm nasal to the optic disc, the CT value of NC was (13.02 ± 0.35μm), the CT value of LIM was (9.60 ± 0.28μm), *t* = 7.62, *P* < 0.01; at 2000μm nasal to the optic disc, the CT value of NC was (13.46 ± 0.51μm), the CT value of LIM was (9.72 ± 0.41μm), *t* = 5.70, *P* < 0.01; a*t* 3000μm nasal to the optic disc, the CT value of NC was (13.98 ± 0.30μm), the CT value of LIM was (9.87 ± 0.52μm), *t* = 6.81, *P* < 0.01; at 1000μm *t*emporal to the optic disc, the CT value of NC was (13.06 ± 0.43μm), the CT value of LIM was (9.97 ± 0.36μm), *t* = 5.51, *P* < 0.01; at 2000μm temporal *t*o the optic disc, the CT value of NC was (13.14 ± 0.45μm), the CT value of LIM value was (9.42 ± 0.30μm), *t* = 6.88, *P* < 0.01; at 3000μm temporal to *t*he optic disc, the CT value of NC was (13.26 ± 0.37μm), the CT value of LIM value was (9.75 ± 0.43μm), *t* = 6.21, *P* < 0.01).

**Fig 7:**
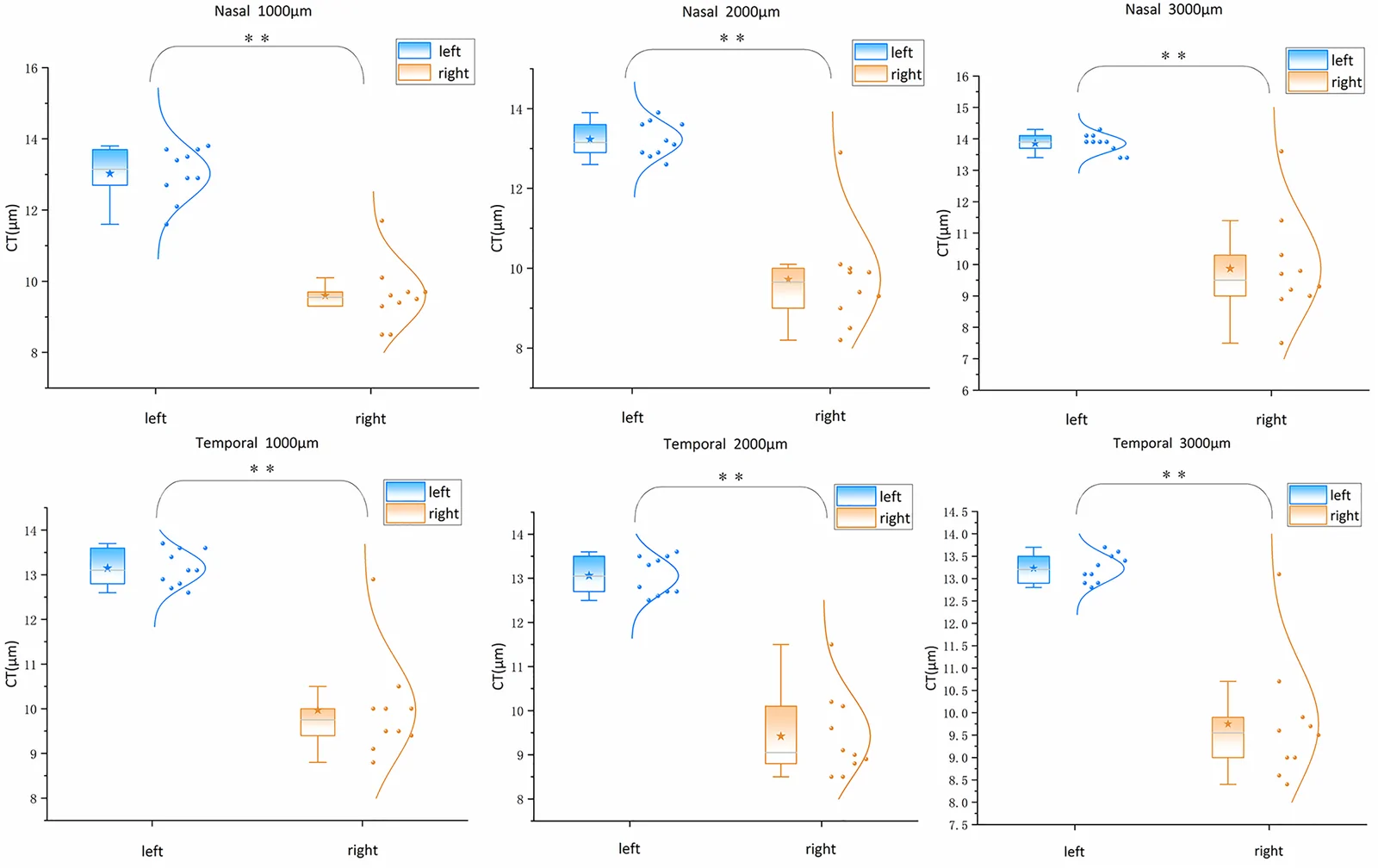
Comparison of CT in LIM between left eye and right eye(μm). (At 1000μm nasal to the optic disc, the CT value of left eye in LIM was (13.03 ± 0.23μm), *t* = 9.34, *P* < 0.01; at 2000μm nasal to the optic disc, the CT value of left eye in LIM was (13.23 ± 0.14μm), *t* = 8.13, *P* < 0.01; a*t* 3000μm nasal to the optic disc, the CT value of left eye in LIM was (13.86 ± 0.09μm), *t* = 7.52, *P* < 0.01; at 1000μm *t*emporal to the optic disc, the CT value of left eye in LIM was (13.15 ± 0.13μm), *t* = 8.30, *P* < 0.01; at 2000μm temporal *t*o the optic disc, the CT value of left eye in LIM was (13.06 ± 0.14μm), *t* = 11.05, *P* < 0.01; at 3000μm temporal to *t*he optic disc, the CT value of left eye in LIM was (13.23 ± 0.10μm), *t* = 7.91, *P* < 0.01).

Compared with NC, the average CT of right eye in LIM rats at each measurement point was significantly thinner, with statistical differences (*t* = 5.51 ~ 7.62, *P* < 0.01). The average CT of right eye in NC rats at six points measured (13.32 ± 0.16) μm, while that in LIM was (9.72 ± 0.15) μm, with a statistically significant difference (*t* = 15.90, *P* < 0.0001). Compared wi*t*h the left eye of LIM, the average CT of right eye in LIM rats at each measurement point was significantly thinner, with statistical differences (*t* = 7.52 ~ 11.05, *P* < 0.01). These findings are depicted in Figs 5, 6 and 7.

**Fig 8:**
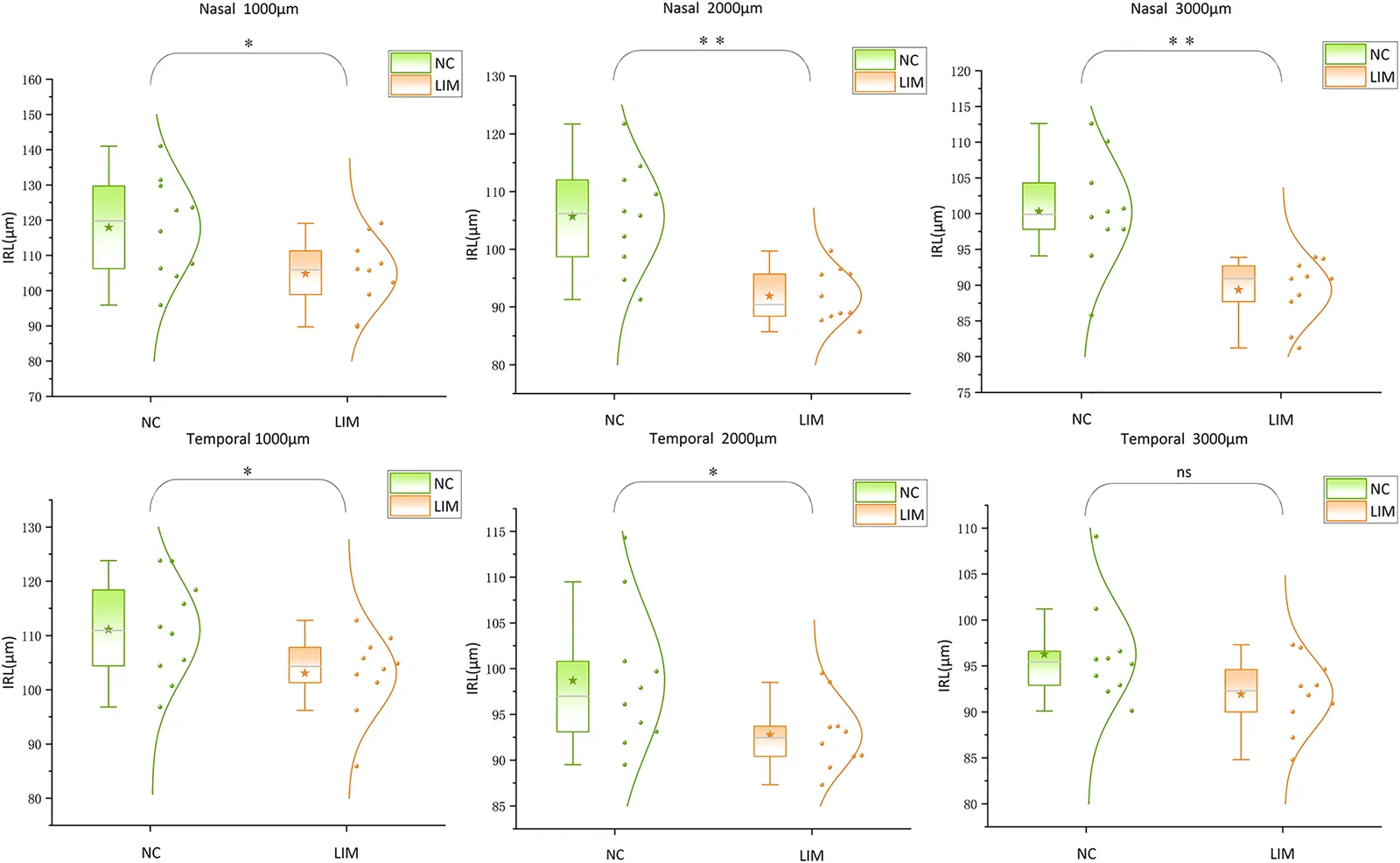
Comparison of IRL thickness of right eye between NC and LIM(μm). (At 1000μm nasal to the optic disc, the IRL value of NC is (117.92 ± 4.51μm), and of LIM it is (104.85 ± 3.16μm), *t* = 2.37, *P* < 0.05; at 2000μm nasal to the optic disc, the IRL value of NC is (105.69 ± 2.94μm), and of LIM it is (91.92 ± 1.48μm), *t* = 4.19, *P* < 0.01; a*t* 3000μm nasal to the optic disc, the IRL value of NC is (100.30 ± 2.42μm), and of LIM it is (89.35 ± 1.39μm), *t* = 3.93, *P* < 0.01; at 1000μm *t*emporal to the optic disc, the IRL value of NC is (111.10 ± 2.95μm), and of LIM it is (103.07 ± 2.39μm), *t* = 2.11, *P* < 0.05; at 2000μm temporal *t*o the optic disc, the IRL value of NC is (98.69 ± 2.49μm), and of LIM it is (92.76 ± 1.22μm), *t* = 2.14, *P* < 0.05; at 3000μm temporal to *t*he optic disc, the IRL value of NC is (96.27 ± 1.71μm), and of LIM it is (91.93 ± 1.25μm), *t* = 2.05, *P* > 0.05).

**Fig 9:**
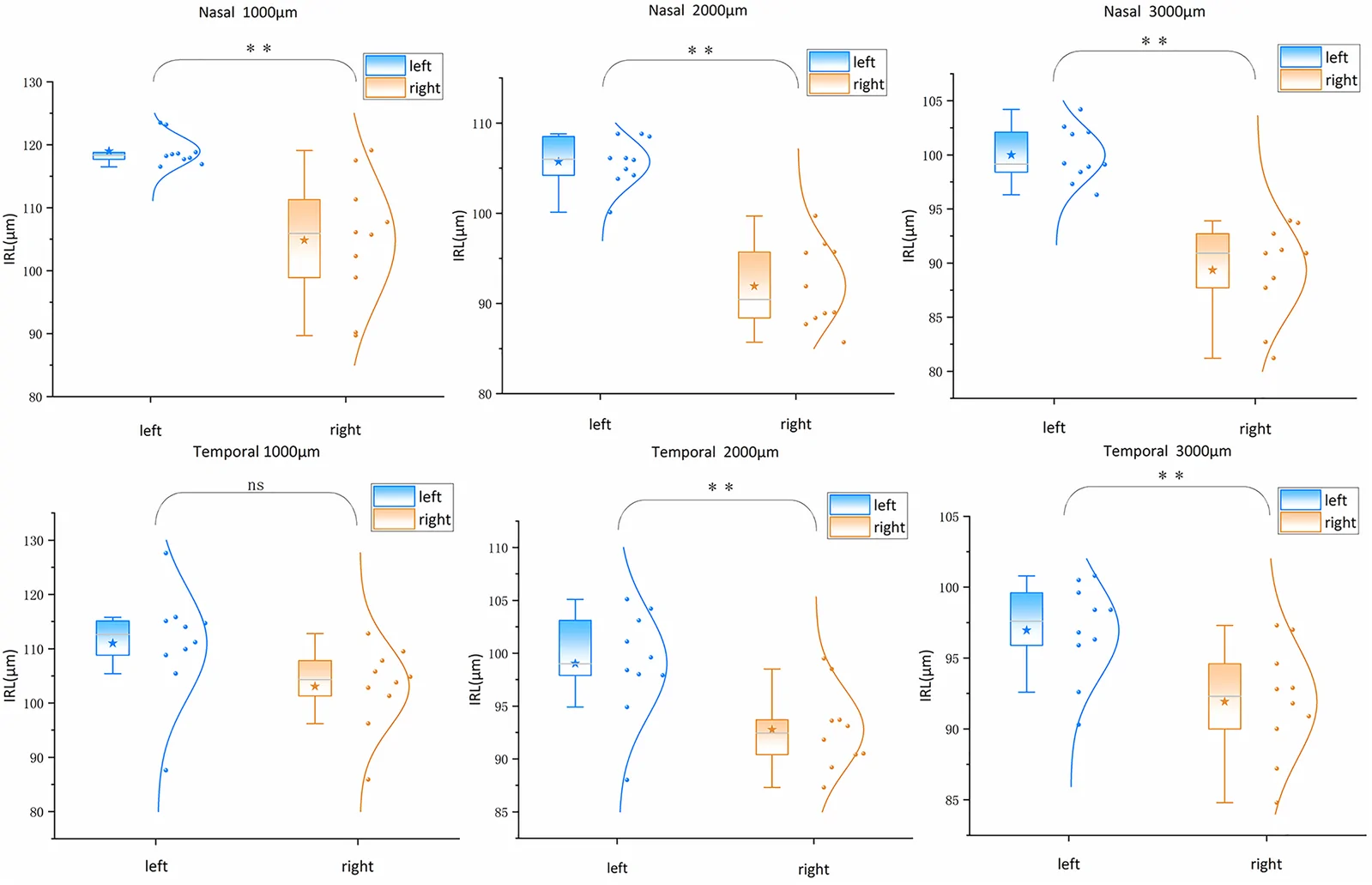
Comparison of IRL thickness in LIM between left eye and right eye(μm). (At 1000μm nasal to the optic disc, the IRL value of left eye in LIM was (118.98 ± 0.76μm), *t* = 4.34, *P* < 0.01; at 2000μm nasal to the optic disc, the IRL value of left eye in LIM was (105.72 ± 0.85μm), *t* = 8.09, *P* < 0.01; a*t* 3000μm nasal to the optic disc, the IRL value of left eye in LIM was (100.00 ± 0.81μm), *t* = 6.64, *P* < 0.01; at 1000μm *t*emporal to the optic disc, the IRL value of left eye in LIM was (101.01 ± 3.20μm), *t* = 1.99, *P* > 0.05; at 2000μm temporal *t*o the optic disc, the IRL value of left eye in LIM was (99.03 ± 1.59μm), *t* = 3.13, *P* < 0.01; at 3000μm temporal to *t*he optic disc, the IRL value of left eye in LIM was (96.96 ± 1.07μm), *t* = 3.05, *P* < 0.01).

**Fig 10:**
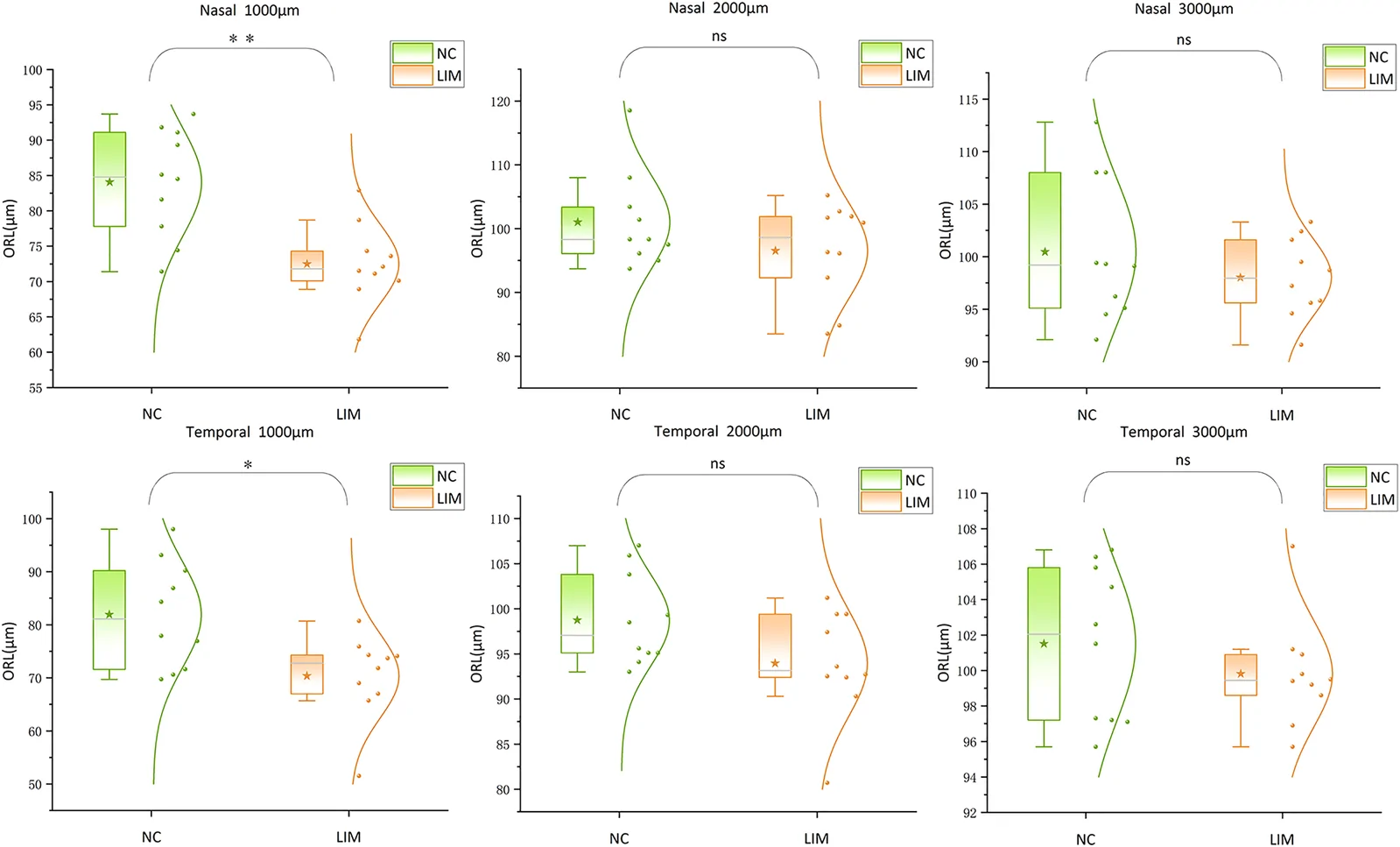
Comparison of ORL thickness of right eye between NC and LIM(μm). (At 1000μm nasal to the optic disc, the ORL value of NC was (84.07 ± 2.43μm), the ORL value of LIM value was (72.50 ± 1.79μm), *t* = 3.84, *P* < 0.01; at 2000μm nasal to the optic disc, the ORL value of NC was (101.02 ± 2.36μm), the ORL value of LIM value was (96.54 ± 2.39μm), *t* = 1.33, *P* > 0.05; a*t* 3000μm nasal to the optic disc, the ORL value of NC was (100.45 ± 2.17μm), the ORL value of LIM value was (98.03 ± 1.19μm), *t* = 0.98, *P* > 0.05; at 1000μm *t*emporal to the optic disc, the ORL value of NC was (81.92 ± 3.18μm), the ORL value of LIM value was (70.37 ± 2.52μm), *t* = 2.85, *P* < 0.05; at 2000μm temporal *t*o the optic disc, the ORL value of NC was (98.74 ± 1.62μm), the ORL value of LIM value was (93.96 ± 1.88μm), *t* = 1.92, *P* > 0.05; at 3000μm temporal to *t*he optic disc, the ORL value of NC was (101.51 ± 1.38μm), the ORL value of LIM value was (99.82 ± 0.96μm), *t* = 1.01, *P* > 0.05).

**Fig 11:**
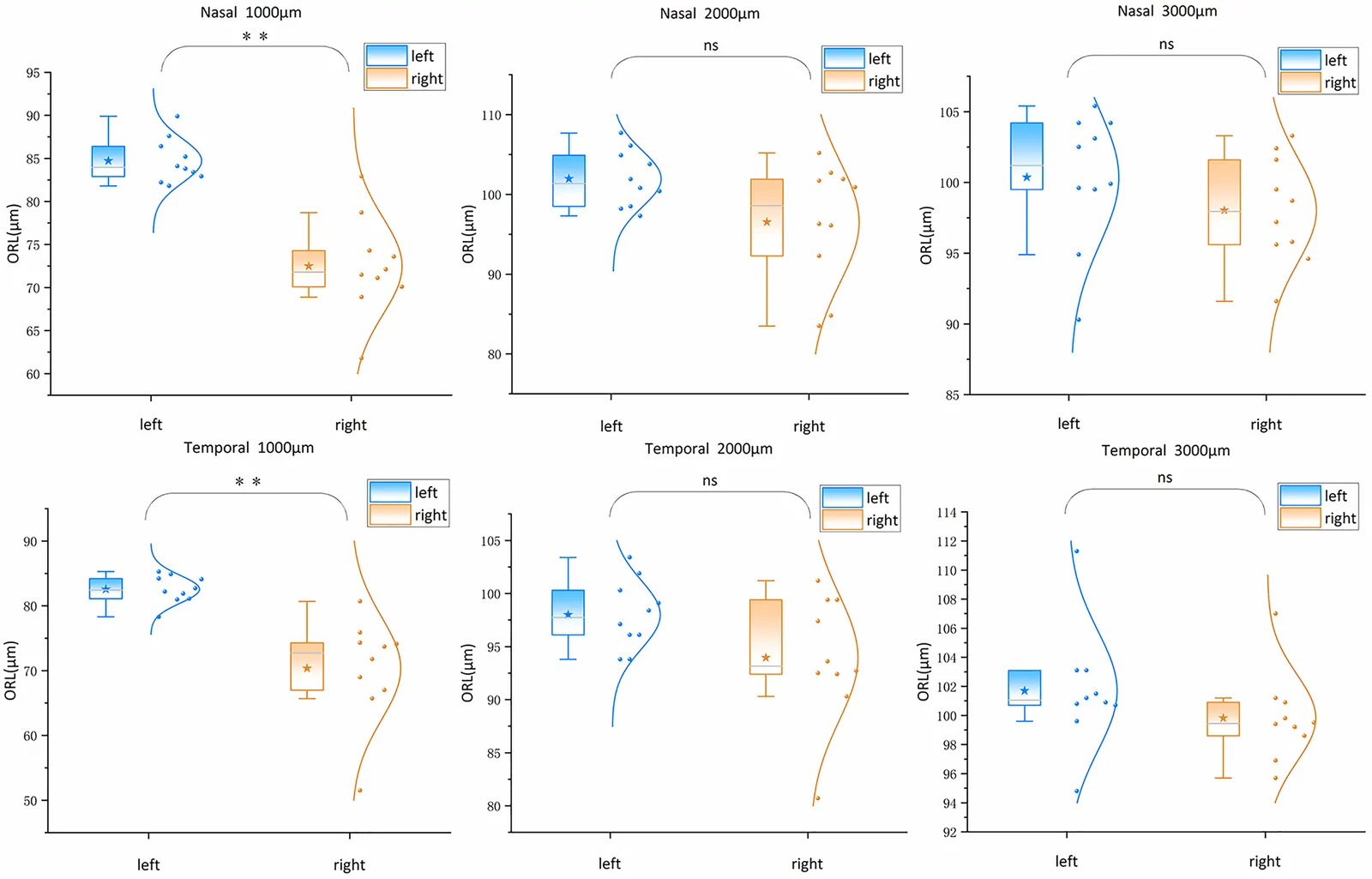
Comparison of ORL thickness in LIM between left eye and right eye(μm). (At 1000μm nasal to the optic disc, the ORL value of left eye in LIM was (84.73 ± 0.81μm), *t* = 6.23, *P* < 0.01; at 2000μm nasal to the optic disc, the ORL value of left eye in LIM was (101.96 ± 1.12μm), *t* = 2.05, *P* = 0.05; a*t* 3000μm nasal to the optic disc, the ORL value of left eye in LIM was (100.36 ± 1.49μm), *t* = 1.22, *P* > 0.05; a*t* 1000μm temporal to the optic disc, the ORL value of left eye in LIM was (82.57 ± 0.68μm), *t* = 4.68, *P* < 0.01; at 2000μm *t*emporal to the optic disc, the ORL value of left eye in LIM was (98.00 ± 1.02μm), *t* = 1.88, *P* > 0.05; at 3000μm *t*emporal to the optic disc, the ORL value of left eye in LIM was (101.70 ± 1.29μm), *t* = 1.17, *P* > 0.05).

**Fig 12:**
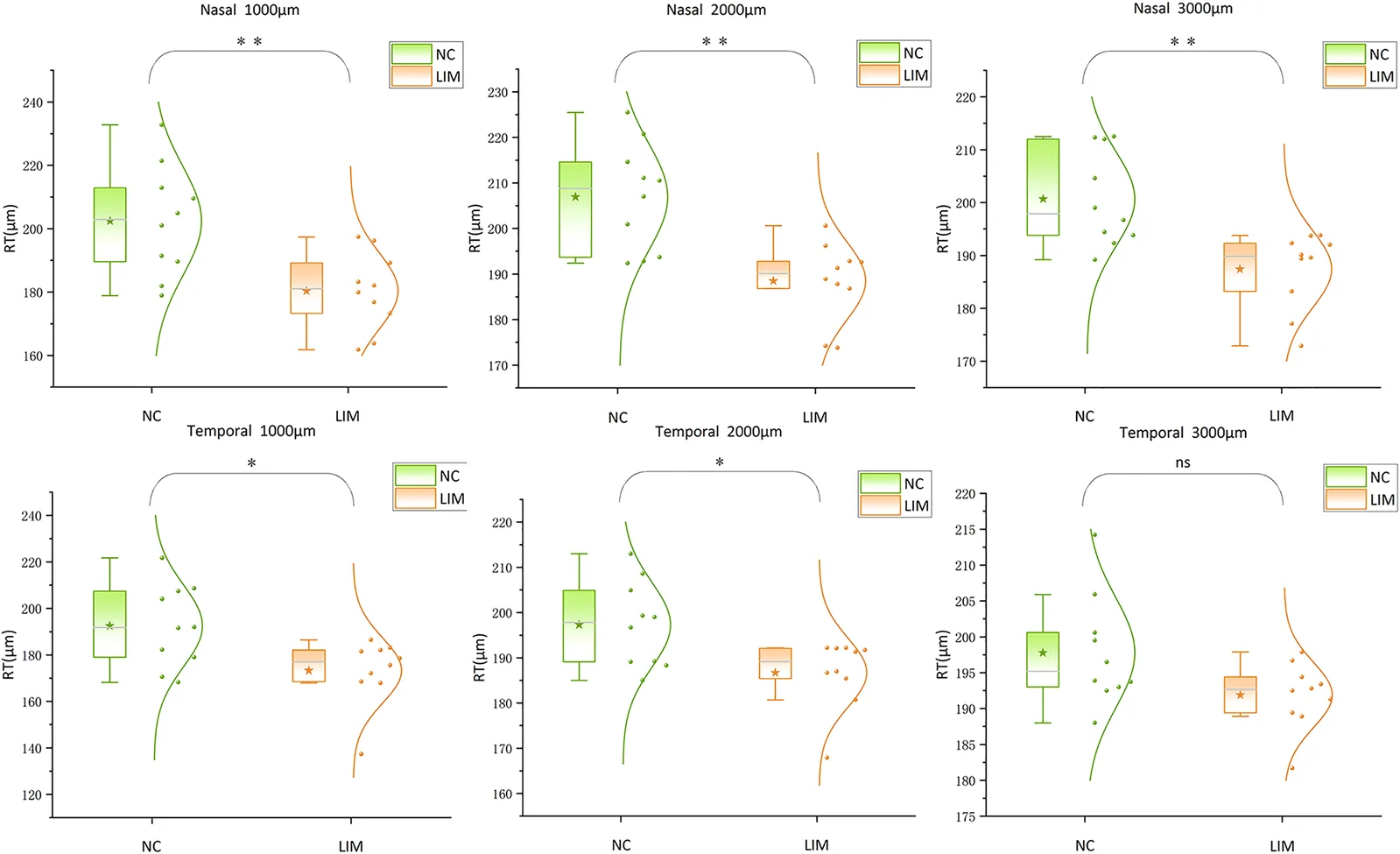
Comparison of RT of right eye between NC and LIM(μm). (At 1000μm nasal to the optic disc, the RT value of NC is (202.43 ± 5.49μm), the RT value of LIM is (180.37 ± 3.82μm), *t* = 3.30, *P* < 0.01; at 2000μm nasal to the optic disc, the RT value of NC is (206.92 ± 3.72μm), the RT value of LIM is (188.50 ± 2.73μm), *t* = 3.99, *P* < 0.01; a*t* 3000μm nasal to the optic disc, the RT value of NC is (200.68 ± 2.84μm), the RT value of LIM is (187.41 ± 2.30μm), *t* = 3.63, *P* < 0.01; at 1000μm *t*emporal to the optic disc, the RT value of NC is (192.53 ± 5.60μm), the RT value of LIM is (173.31 ± 4.47μm), *t* = 2.68, *P* < 0.05; at 2000μm temporal *t*o the optic disc, the RT value of NC is (197.31 ± 2.99μm), the RT value of LIM is (186.72 ± 2.42μm), *t* = 2.75, *P* < 0.05; at 3000μm temporal to *t*he optic disc, the RT value of NC is (197.78 ± 2.42μm), the RT value of LIM is (191.90 ± 1.45μm), *t* = 2.09, *P* > 0.05).

**Fig 13:**
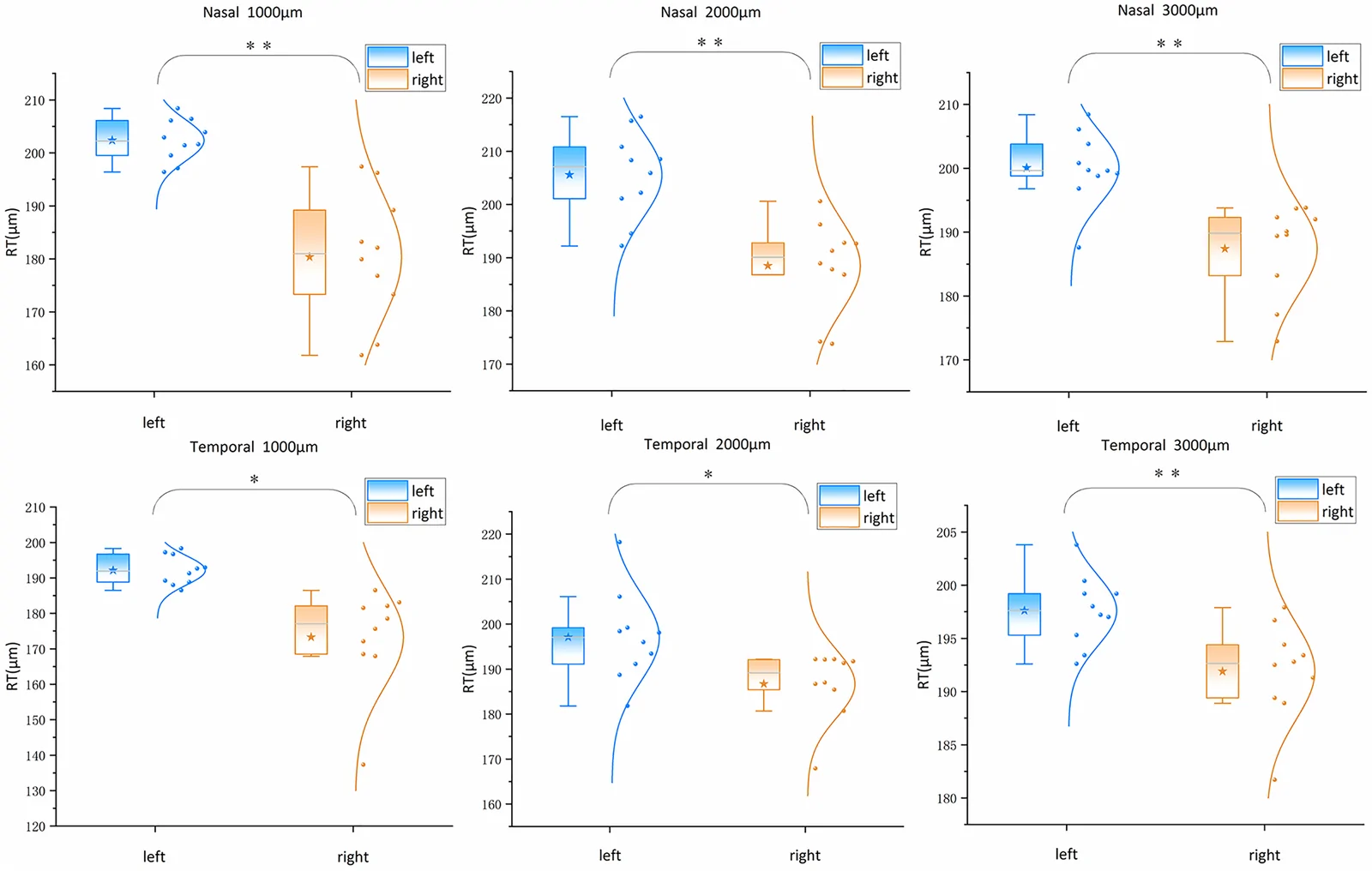
Comparison of RT in LIM between left eye and right eye(μm). (At 1000μm nasal to the optic disc, the RT value of left eye in LIM was (202.37 ± 1.26μm), *t* = 5.47, *P* < 0.01; at 2000μm nasal to the optic disc, the RT value of left eye in LIM was (205.57 ± 2.58μm), *t* = 4.54, *P* < 0.01; a*t* 3000μm nasal to the optic disc, the RT value of left eye in LIM was (200.08 ± 1.79μm), *t* = 4.35, *P* < 0.01; at 1000μm *t*emporal to the optic disc, the RT value of left eye in LIM was (192.15 ± 1.31μm), *t* = 4.05, *P* < 0.05; at 2000μm temporal *t*o the optic disc, the RT value of left eye in LIM was (197.10 ± 3.14μm), *t* = 2.62, *P* < 0.05; at 3000μm temporal *t*o the optic disc, the RT value of left eye in LIM was (197.61 ± 1.05μm), *t* = 3.17, *P* < 0.01).

The average thickness of the IRL of right eye at each measurement point in LIM rats was thinner compared with NC, with statistical differences at all measurement points except at 3000μm on the temporal side (*t* = 2.11~ 4.19, *P* < 0.05). The average thickness of the ORL of right eye at each measurement point in LIM rats was thinner compared with NC, with statistical differences only at 1000μm on the nasal and temporal sides (*t* = 3.84, *P* < 0.01; *t* = 2.85, *P* = 0.0106). The average thickness of *t*he RT of right eye at each measurement point in LIM rats was thinner compared with NC, with statistical differences at all measurement points except at 3000μm on the temporal side (*t* = 2.68 ~ 3.99, *P* < 0.05). Compared with *t*he left eye of LIM, the average IRL of right eye in LIM rats at each measurement point was thinner, with statistical differences at all measurement points except at 1000μm on the temporal side (*t* = 3.05 ~ 8.09, *P* < 0.01). Compared with the lef*t* eye of LIM, the average ORL of right eye in LIM rats was thinner, with statistical differences only at 1000μm on the nasal and temporal sides (*t* = 6.23, *P* < 0.01; *t* = 4.68, *P* < 0.01). Compared with the lef*t* eye of LIM, the average RT of righ*t* eye in LIM rats at each measurement point was thinner, with statistical differences at all measurement points (*t* = 2.62 ~ 5.47, *P* < 0.05). These findings are depicted in Figs 5 and 8–13.

### Retinal inner layer blood flow changes

**Fig 14:**
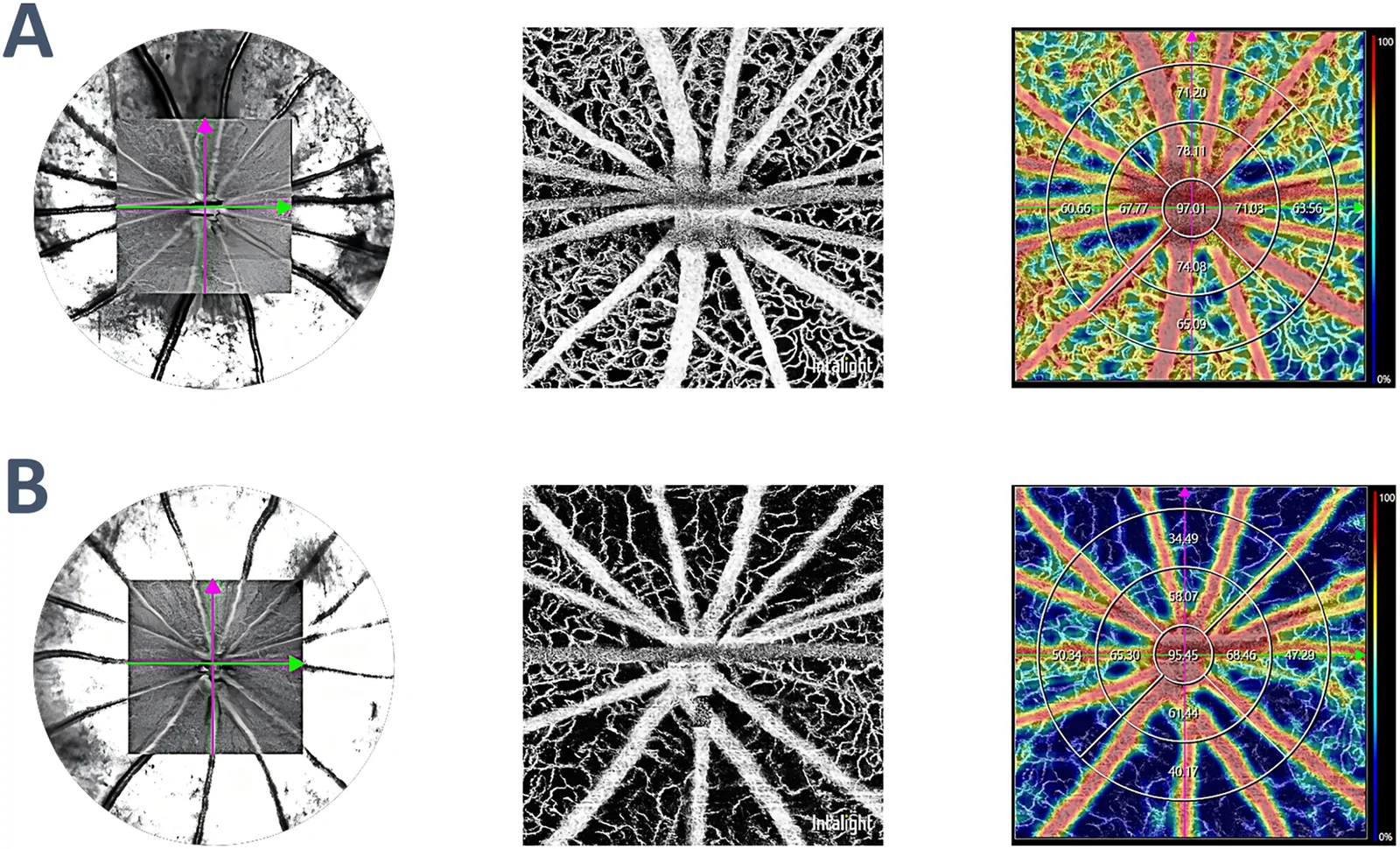
Quantitative map of blood flow in the inner layer of the rat retina. (Fig 14-A is for NC rats, Fig 14-B is for LIM rats).

**Fig 15:**
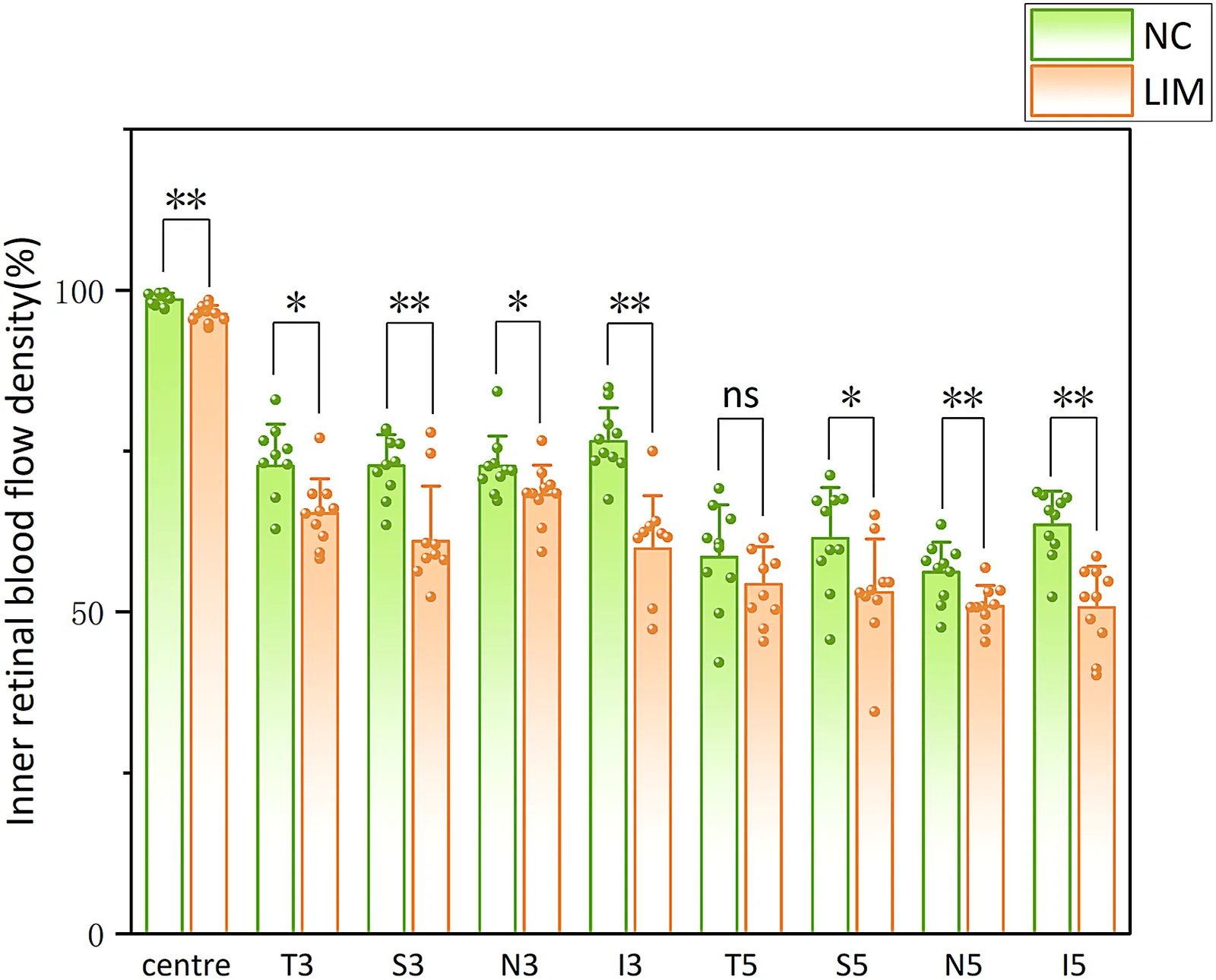
Comparison of blood flow density in the inner retinal layers of right eye between NC and LIM(%). (The blood flow density of NC in the central area is (98.52 ± 0.32%), LIM is (96.28 ± 0.43%), *t* = 4.21, *P* < 0.01; the blood flow density of NC in T3 is (72.68 ± 2.05%), LIM is (65.34 ± 1.69%), *t* = 2.77, *P* < 0.05; *t*he blood flow density of NC in S3 is (72.71 ± 1.54%), LIM is (61.00 ± 2.71%), *t* = 3.76, *P* < 0.01; the blood flow density of NC in N3 is (72.66 ± 1.48%), LIM is (68.23 ± 1.45%), *t* = 2.13, *P* < 0.05; the blood flow density of NC in I3 is (76.52 ± 1.64%), LIM is (59.84 ± 2.60%), *t* = 5.43, *P* < 0.01;the blood flow density of NC in T5 is (58.56 ± 2.55%), LIM is (54.32 ± 1.84%), *t* = 1.35, *P* > 0.05; the blood flow density of NC in S5 is (61.47 ± 2.49%), LIM is (53.06 ± 2.62%), *t* = 2.33, *P* < 0.05; the blood flow densi*t*y of NC in N5 is (56.19 ± 1.47%), LIM is (50.89 ± 1.01%), *t* = 2.97, *P* < 0.01; the blood flow density of NC in I5 is (63.57 ± 1.64%), LIM is (50.74 ± 2.01%), *t* = 4.94, *P* < 0.01).

**Fig 16:**
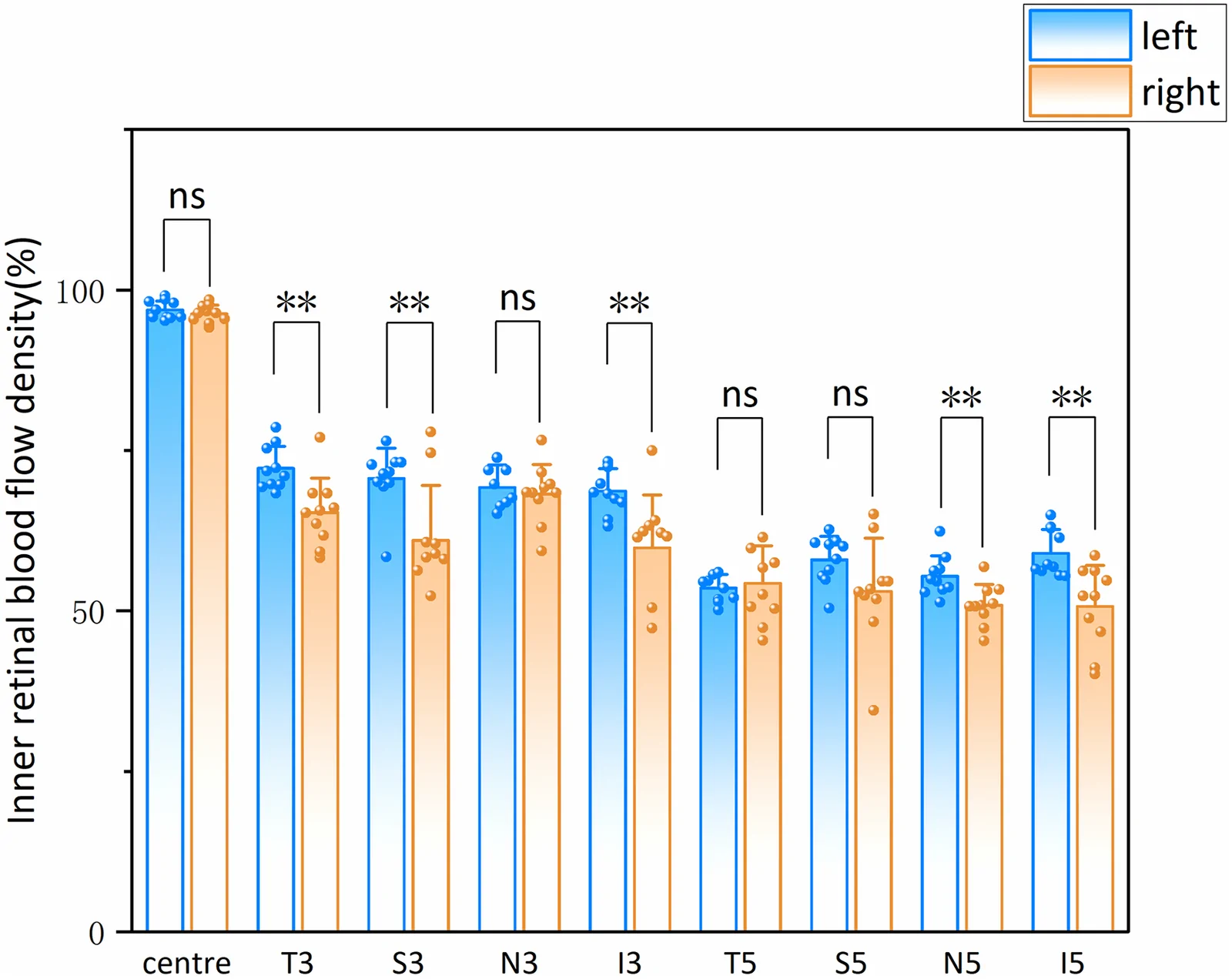
Comparison of blood flow density in the inner retinal layers in LIM between left eye and right eye(μm). (The blood flow density of left eye in LIM in the central area is (96.87 ± 0.46%), *t* = 0.95, *P* > 0.05; the blood flow density of left eye in LIM in T3 is (72.23 ± 1.08%), *t* = 3.43, *P* < 0.01; *t*he blood flow density of left eye in LIM in S3 is (70.64 ± 1.50%), *t* = 3.11, *P* < 0.01; the blood flow density of left eye in LIM in N3 is (69.26 ± 1.10%), *t* = 0.57, *P* > 0.05; the blood flow density of left eye in LIM in I3 is (68.67 ± 1.10%), *t* = 3.13, *P* < 0.01;the blood flow density of left eye in LIM in T5 is (53.57 ± 0.67%), *t* = 0.38, *P* > 0.05; the blood flow density of left eye in LIM in S5 is (57.96 ± 1.17%), *t* = 1.71, *P* > 0.05; *t*he blood flow density of left eye in LIM in N5 is (55.42 ± 1.00%), *t* = 3.18, *P* < 0.01; the blood flow densi*t*y of left eye in LIM in I5 is (59.02 ± 1.15%), *t* = 3.57, *P* < 0.01).

Compared with NC, the blood flow density in the inner retinal layers of each partition of right eye in LIM is reduced, with no statistically significant difference in the T5 area (*t* = 1.35, *P* = 0.20), but there are statistically significant differences in the remaining partitions (*t* = 2.13 ~ 5.43, *P* < 0.05). Compared with the left eye of LIM, the blood flow density in the inner retinal layers of each partition of right eye in LIM is reduced, with statistical differences only in the S3, T3, I3, I5 and N5 area (*t* = 3.11, *P* < 0.01; *t* = 3.43, *P* < 0.01; *t* = 3.13, *P* < 0.01; *t* = 3.57, *P* < 0.01; *t* = 3.18, *P* < 0.01). Refer *t*o Figs 14, 15,16 for further de*t*ails.

## Discussion

The specific mechanism of myopia onset is not yet clear, but current research suggests that with the increase in myopia degree, there will be pathological changes such as axial elongation, thinning of the retinal pigment epithelium, thinning of the choroid, myopic crescent, lacquer cracks, and atrophic lesions of the retina and choroid [7]. The choroid is a vascular layer located between the sclera and the Bruch’s membrane, and increasing evidence indicates that the choroid is involved in the regulation of eye growth, potentially influencing the development of myopia and its treatment. As early as 1995, Wallman et al [8] discovered that in a myopic chick model, the CT was regulated by retinal defocus to compensate for the applied refractive error, and these alterations were related to variations in eye growth. Numerous animal studies have demonstrated that compared to eyes with normal vision, the choroid is thinner in myopic eyes and thicker in hyperopic eyes [9,10]. In related clinical studies, it has also been confirmed that adult myopic patients are accompanied by significant choroidal thinning [11,12]. During the development of myopic children, the choroid thins rapidly over time, while in non-myopic children, the CT increases significantly over time [13,14]. The thinning of the choroid is often accompanied by axial elongation, suggesting that changes in CT may be a predictive biomarker for long-term changes in AL [5]. Thinning of the choroid is a structural feature of myopia, and CT is negatively correlated with AL. Studies suggest that the choroid may secrete growth factors that stimulate or inhibit the growth of the sclera, participating in blood vessel formation and regulating the remodeling of the scleral extracellular matrix [15]. Consequently, alterations in CT are also related to changes in scleral growth and may play a crucial role in the homeostatic control of eye growth [16,17].

To conduct choroidal-related research to explore the etiology and pathogenesis of myopia development and attempt to intervene in the occurrence and development of myopia with drugs and other measures, we must first validate this in animal models of myopia. At present, commonly used myopia models include form deprivation and defocus, typically utilizing experimental animals such as rats, guinea pigs, chickens, and monkeys, etc. Among them, rats have emerged as significant and commonly used myopia model animals due to their gentle temperament, favorable response to myopia modeling, and the convenience of genetic editing as model animals.

The utilization of OCTA to measure ocular blood flow and the thickness of choroid and retina has yielded numerous clinical practice successes. As a sophisticated fundus imaging technology, it can qualitatively assess and quantitatively analyze alterations in the macula, optic disc, choroid, retina, and sclera, allowing clinicians to obtain high-resolution images of even the deepest ocular structures [18,19].OCTA technology has also emerged as a vital and commonly used method for observing choroidal and retinal structures in current myopia research. Although certain studies have used OCT to observe alterations in myopic mouse parameters such as curvature radius, lens thickness, vitreous cavity depth, and pupil width [20], there are currently no studies using OCTA to measure choroidal and retinal thickness and blood flow changes in a lens-induced myopic rat model. Therefore, in this study, we used OCTA to measure the AL, choroidal and retinal layer thickness in a rat model of lens-induced myopia, quantified changes in retinal inner layer blood flow density. Through dual validation involving intergroup comparisons (LIM vs. NC) and intra-group self-control comparisons (LIM right eye vs. LIM left eye), our aim was to provide a scientific basis for in-depth research on choroidal and retinal thickness and blood flow changes in the lens-induced myopic rat model, as well as exploration of related mechanisms in the etiology and pathogenesis of myopia.

The results of this study indicate that the CT of right eye in LIM rats is thinner than that of NC, exhibiting a statistically significant difference, and the most pronounced thinning is at 1000μm nasal to the optic disc, which is consistent with the outcomes of previous animal experiments and human research. The self-control results further validated this finding: the CT of the right eye in LIM rats was significantly thinner at all measurement points compared to that of the left eye, with the difference being statistically significant. No significant difference in CT was observed between the left eye (self-control) and the right eye of NC rats (normal control), strongly ruling out interference from individual growth factors or systemic influences on the experimental results and confirming that choroidal thinning is indeed a specific alteration induced by lens-induced local optical defocus. Some studies have also found that during the process of human myopia, the choroid in the nasal region is the first to exhibit thinning, and the changes become increasingly pronounced with the development of myopia [21]. This may suggest that rats, like humans, during the process of lens-induced myopia, the first area to exhibit choroidal thinning is also the nasal side.

The inner, outer, and entire thickness of the LIM retina of right eye are thinner than those of the NC retina, and the inner retinal blood flow density of LIM is lower than that of NC. This phenomenon could be attributed to the mechanical traction resulting from the continuous extension of the eyeball axis during the development of myopia, which causes alterations in RT and inflicts harm upon the inner retinal blood vessels, leading to a decrease in central retinal artery blood flow velocity, a narrowing of retinal vessel diameter, and a decrease in capillary density [22–24].This impacts the blood supply and metabolism of the ORL, leading to insufficient nutrition and oxygen supply, a decrease in retinal ganglion cell density, a reduction in the number of nuclear cell layers, and a gradual atrophy and thinning of the retina, resulting in irreversible vision loss [22,25,26].

The results of the study show that there is no statistically significant difference between the two groups in the thickness of the IRL and the RT at 3000μm temporal to the optic disc, and in the blood flow density of the inner retinal layer in the T5 area. This may be due to the fact that with the development of myopia, retinal vascular remodeling occurs, with an increase in the number of capillaries and the density of branching points [27].

This phenomenon may first appear in the temporal region, leading to a compensatory increase in RT, which yields no statistically significant difference from normal eyes. Self-control analysis further refined this finding: in LIM rats, the IRL and RT of the right eye were lower than those of the left eye at all measurement points; however, the difference in IRL at 1000 μm temporal distance was not statistically significant, consistent with the absence of significant differences in IRL and RT at 3000 μm temporal distance in intergroup comparisons. For ORL, differences were confined to the 1000 μm parapapillary region (nasal and temporal sides) in both intergroup comparisons and self-control analyses, suggesting that retinal thinning in the outer layer may first occur in the paracentral area during early myopia. These data collectively delineate the “spatiotemporal characteristics” of myopia-induced retinal changes: alterations in the inner layer are more extensive, while those in the outer layer are relatively limited, and compensatory responses may be present in the temporal region. Nevertheless, some studies have found that myopic eyes may show thinning of the IRL, but no difference in the thickness of the ORL [28]. This difference may be due to different myopic animal models used in the studies. The effects of myopia on each layer of the retina and its specific mechanisms require further in-depth research in the future.

This study used OCTA technology to scan the choroid and retina of lens-induced myopic rats and found that the CT of myopic rats became thinner, along with the thickness of both the inner and outer retinal layers, as well as the RT. Additionally, the density of inner retinal blood flow was observed to decrease. These findings are consistent with the results of human myopia research. This validates the feasibility of the lens-induced rat model and provides a theoretical basis and foundation for rats as model animals for myopia-related research, as well as for research on the mechanisms of choroidal and retinal-related myopia onset.While it yielded informative results, several limitations should be acknowledged. The modeling and observation period of the experiment was 8 weeks. While this duration is sufficient to reflect acute or subacute changes following myopia induction, it cannot reveal the long-term dynamic progression of choroidal and retinal thinning as well as reduced blood flow, or determine whether these changes are reversible or will continue to progress.The study employed only a -5D lens-induced defocus myopia model. Choroidal and retinal alterations may differ in models established with different degrees of defocus or alternative methods such as form deprivation. Therefore, the general applicability of the conclusions requires further validation under different modeling parameters.Based on these limitations, our future research will focus on the following directions:First,increasing the sample size and extending the observation period are needed to investigate the long-term progression and potential reversibility of the observed choroidal and retinal changes.Second,utilizing different defocus degrees and alternative induction methods should be conducted to validate and compare the patterns of structural and vascular alterations, enhancing the generalizability of our findings.

## Supporting information

S1 File Minimal dataset used for the analyses in this study. (XLSX)

## Abbreviations

AL: axial length
ACS: anterior corneal surface
CT: choroidal thickness
ILM: inner limiting membrane
IRL: inner retinal layer
LIM: lens-induced myopia
NC: normal control
OCTA: optical coherence tomography angiography
ORL: outer retinal layer
RPE: retinal pigment epithelium
RT: retinal thickness
